# The Management of Coronary Artery Disease in TAVR Patients

**DOI:** 10.3390/jcm12227126

**Published:** 2023-11-16

**Authors:** Laurent Faroux, Aurélien Villecourt, Damien Metz

**Affiliations:** Cardiology Department, Reims University Hospital, 51100 Reims, France

**Keywords:** coronary artery disease, TAVR, fractional flow reserve, coronary computed tomography angiography

## Abstract

About half of the transcatheter aortic valve replacement (TAVR) recipients exhibit some degree of coronary artery disease (CAD), and controversial results have been reported regarding the impact of the presence and severity of CAD on clinical outcomes post-TAVR. In addition to coronary angiography, promising data has been recently reported on the use of both cardiac computed tomography angiography and the functional invasive assessment of coronary lesions whether by FFR or iFR in the work-up pre-TAVR. Despite mitigated available data, percutaneous revascularization of significant coronary lesions has been the routine strategy in TAVR candidates with CAD. Additionally, scarce data exists on the incidence, characteristics and management of coronary events post-TAVR, and increasing interest exists on the potential coronary access challenges in patients requiring coronary angiography/intervention post-TAVR. This review provides an updated overview of the knowledge of CAD in TAVR recipients, focusing on its prevalence, clinical impact, pre- and post-procedural evaluation and management.

## 1. Introduction

**Figure 1:**
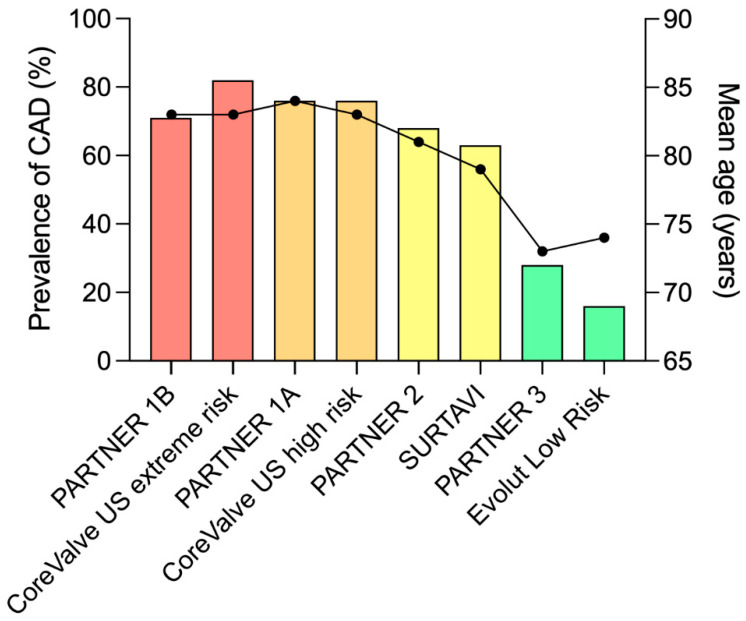
Prevalence of coronary artery disease in randomized trials. CAD: coronary artery disease. Prevalence of CAD (bars), mean age (bullets).

Aortic stenosis and coronary artery disease (CAD) share some common risk factors, and some studies have suggested a similar pathophysiology process for both entities [1]. About half of transcatheter aortic valve replacement (TAVR) candidates included in observational registries exhibit some degree of CAD [2]. In fact, the prevalence of CAD depends largely on the population studied, and within randomized trials the prevalence of CAD decreases from 81% to 15% as the mean age and surgical risk of patients decreases [3,4,5,6,7,8,9,10] (Figure 1). When present, CAD is multi-vessel in half of the cases and involves the left main stem in 10% of the patients [2].

## 2. Prognostic Impact of CAD in TAVR Candidates

Numerous observational studies have aimed to assess the prognostic impact of CAD in TAVR recipients and reported conflicting results. These studies have been pooled in several meta-analyses concluding that coronary artery disease and even more its degree of severity as estimated by the SYNTAX score were markers of poor prognosis in this population. However, CAD is often associated with other comorbidities and when statistical adjustment was performed, the negative prognostic impact of CAD was no longer significant [2]. Thus, it seems likely that CAD and its severity are more markers of comorbidity and high surgical risk than independent factors of poor prognosis.

## 3. Coronary Evaluation Pre-TAVR

Coronary angiography is the gold standard for the identification of coronary stenosis, either before cardiac surgery or percutaneous procedures [11]. Nevertheless, the use of cardiac computed tomography angiography (CCTA) in the setting of pre-TAVR work-up has shown promising preliminary data [12]. On the other hand, the use of hemodynamic assessment of coronary lesions by FFR (fractional flow reserve) or iFR (instantaneous wave-free ratio) in patients with aortic stenosis is safe and reliable and is an emerging strategy in this context [2].

Some authors have proposed that pre-TAVR coronary angiography could only be performed when CCTA suggests the presence of significant coronary stenosis. Thus, an early study concluded that coronary angiography was only necessary for a minority of TAVR candidates, with no negative clinical impact associated with the avoidance of coronary angiogram based on CCTA results [13]. Numerous studies have since sought to investigate the performance of CCTA in this indication, showing an excellent negative predictive value at the cost of low specificity [12]. Assessment of previously stented segments was feasible in 70 to 90% of cases with a good diagnosis performance [2]. Conversely, the rate of false positives increased when heavily calcified segments were evaluated. Finally, a meta-analysis estimated that the performance of CCTA would reduce the number of pre-TAVR coronary angiograms by 37% [12], and this percentage would likely increase as TAVR indication will be increased in younger patients with a much lower probability of CAD and a lower degree of coronary calcification.

Most TAVR candidates do not present with angina pectoris nor have recent non-invasive ischemia testing. Therefore, in accordance with current guidelines, hemodynamic evaluation by FFR or iFR should be performed when the degree of coronary stenosis is less than 90% [14]. However, patients with aortic stenosis were not represented in FFR studies, and scarce data are available on the functional evaluation of coronary lesions in TAVR candidates. The safety of adenosine injection (whether intravenous or intracoronary) has been well demonstrated in several studies [2]. However, it should be noted that nitrate injection was only performed in a minority of cases. Moreover, left ventricular hypertrophy induced by aortic stenosis likely alters the coronary reserve, which could tamper with the results of the hemodynamic assessments. Ahmad et al. [15] demonstrated that systemic flow and coronary hyperemic flow increased significantly after TAVR (compared with pre-TAVR measurements), which could lead to an underestimation of lesion severity when FFR assessment is performed prior to TAVR. In contrast, flow during the wave-free period of diastole did not change after TAVR, suggesting that iFR may not be influenced by the presence of aortic stenosis. Finally, Yamanaka et al. [16] showed, in a population of patients with severe aortic stenosis and coronary lesions previously assessed by myocardial perfusion scintigraphy, that there was a good correlation between FFR and the presence of ischemia on scintigraphy (area under the curve of 0.93). The optimal FFR threshold was 0.83, confirming that FFR likely underestimates lesion severity in the presence of aortic stenosis. These data remain preliminary, and the recent updated guidelines for the management of valvular heart disease still support the use of “classic” thresholds for FFR and iFR (0.80 and 0.89, respectively) in the presence of valvular heart disease [11,17].

## 4. Coronary Revascularization Pre-TAVR

**Figure 2:**
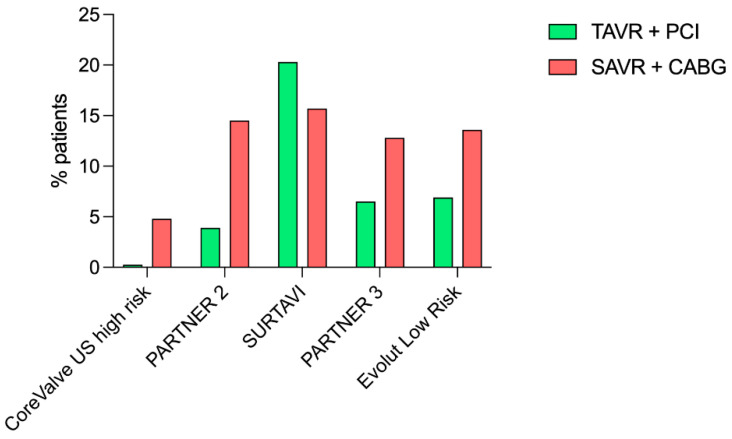
Coronary revascularization in randomized trials. CABG, coronary artery bypass graft; PCI, percutaneous coronary intervention; SAVR, surgical aortic valve replacement; TAVR, transcatheter aortic valve replacement.

Both European and North American guidelines recommend performing percutaneous coronary intervention (PCI) prior to TAVR for coronary stenosis greater than 70% severity in proximal coronary segments, with a recommendation grade of IIa and a level of evidence of C [11,17]. Indeed, only a minority of patients included in randomized trials benefited from coronary revascularization (either by PCI or coronary artery bypass graft) associated with aortic valve replacement (Figure 2), while patients with complex coronary lesions (high SYNTAX score and/or unprotected left main) were excluded from these trials [7,8,9,10]. The largest cohort of patients who underwent pre-TAVR PCI published to date included 1197 patients with a total of 1705 coronary lesions treated and a median follow-up of 2 years post-TAVR [18]. Coronary lesions were frequently complex (bifurcation, ostial location, significant calcifications), with rotational atherectomy used in 7% of patients. Rates of procedural success (97.3%), intra-stent restenosis (2.3%) and stent thrombosis (0.4%) were similar to what is usually described in “all-comers” PCI. The clinical event rate (death, myocardial infarction, and stroke: MACCE) was relatively high, with incomplete coronary revascularization determining an increased risk of MACCE. Witberg et al. [19] found similar results, with patients completely or reasonably incompletely revascularized (residual SYNTAX score ≤8) having a prognosis similar to patients without CAD. In contrast, patients with incomplete coronary revascularization (residual SYNTAX score >8) had a poorer prognosis. On the other hand, the REVASC-TAVI registry [20] did not find any significant impact of the completeness of myocardial revascularization pre-TAVR. ACTIVATION trial [21] randomized patients presenting with aortic stenosis with planned TAVR and significant CAD were split into two arms as follows: PCI versus medical treatment of CAD. Patients who presented an acute coronary syndrome within 1 month before inclusion, as well as those exhibiting severe angina, or an unprotected left main lesion were excluded. A single coronary lesion was revascularized in most cases (71%), and drug-eluting stents were implanted in 80% of patients. After a 12-month follow-up, there was no significant difference between the two strategies regarding the primary endpoint (death or rehospitalization) without reaching the non-inferiority threshold; however, there was a significantly higher rate of bleeding in the PCI arm. However, it should be noted that the authors did not provide information on the use of hemodynamic tools, such as FFR, nor on the completeness of myocardial revascularization, which may have mitigated a potential beneficial effect of pre-TAVR coronary revascularization. We can also assume that a positive effect of pre-TAVR PCI may require a longer follow-up to be shown.

Finally, patients with severe CAD (unprotected left main lesion and/or high SYNTAX score) were excluded from randomized trials (Table 1), and to date only one observational study (with propensity score matching) compared outcomes of a surgical strategy (coronary artery bypass graft and surgical aortic valve replacement) with those of a transcatheter strategy (PCI followed by TAVR) in this specific population [22]. After a median follow-up of 3 years, a similar rate of MACCE was observed between the two strategies. To note, an increased risk of repeat coronary revascularization was observed in patients benefiting from a transcatheter strategy.

In conclusion, the clinical relevance of PCIs performed prior to TAVR remains unclear, and future studies should better identify patients who benefit from pre-TAVR coronary revascularization.

## 5. Timing of Coronary Revascularization Pre-TAVR

When a PCI indication is established, it is usually performed upstream of the TAVR, but the optimal timing of PCI prior to TAVR remains unclear. Two observational studies suggested that PCI performed during the same hospitalization as the TAVR procedure were associated with an increased risk of complications [23,24], while several studies have shown the feasibility and safety of TAVR and concomitant PCI [2]. Although this later strategy has the advantage of avoiding multiple procedures and can potentially reduce the risks associated with obtaining vascular access at different time points, it increases the amount of contrast media administered at the time of the TAVR procedure and could potentially increase the risk of acute kidney injury, particularly in cases of complex CAD. Finally, A sub-analysis of the REVASC-TAVI Registry has been presented at the Cardiovascular Research Technologies (CRT) 2023 and suggested that PCI performed after TAVR showed significantly lower rates of all-cause death and a composite endpoint compared with other PCI timings relative to TAVR. In summary, no definite data exist on the timing of PCI in TAVR candidates. In the absence of further data, no specific timing strategy can be recommended in this setting. However, the presence of both complex CAD and risks factors for contrast nephropathy should probably be considered when establishing a minimum delay between procedures.

## 6. Coronary Management during TAVR

**Figure 3:**
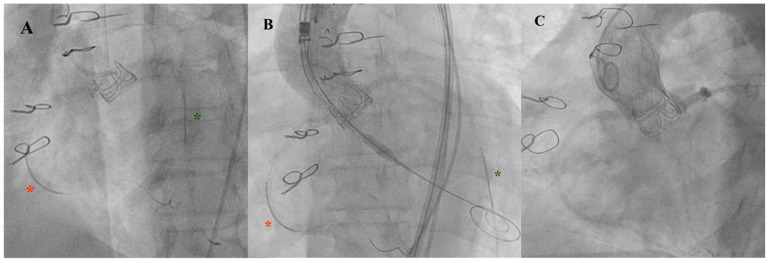
Bilateral coronary protection during valve-in-valve TAVR with high risk of coronary obstruction. Non-expanded coronary stent into right (red asterisk) and left coronary arteries (green asterisk) before (**A**) and during (**B**) valve deployment. Coronary stents were removed at the end of the procedure because no coronary obstruction was observed (**C**).

Coronary artery obstruction is one of the most dreaded procedural complications of TAVR. Distance between coronary ostium and aortic annulus is a major predictor of the risk of coronary occlusion, and the cut-off >10 mm is widely used. Valve-in-valve procedures (i.e., the placement of a TAVR valve within a failed surgical bioprosthesis or a first TAVR valve) are also situations associated with an increased risk. When a high risk of coronary occlusion is identified on the pre-TAVR CT-scan, placement of a non-expanded stent on a guidewire within the coronary artery(s) at risk of occlusion enables immediate action in case of acute coronary occlusion after valve deployment (Figure 3). Finally, when coronary occlusion is considered certain, it is possible to stent the coronary artery with a long protrusion into the aorta during valve deployment (chimney technique).

## 7. Acute Coronary Syndrome after TAVR

Initially neglected because of the advanced age and short life expectancy of first TAVR recipients, the issue of post-TAVR coronary events has progressively become more important and constitutes one of the last limits to the extension of TAVR to patients beyond 75 years. The first study addressing this topic was published in 2017, and it reported that in a single-center cohort of nearly 800 patients with a median follow-up of 2 years post-TAVR the cumulative incidence of post-TAVR ACS was 10% [25]. Male gender, prior CAD, a non-transfemoral approach, and acute kidney injury were independent predictors of post-TAVR ACS occurrence. Several features unique to post-TAVR ACS were then promptly identified. First, ACS following TAVR was characterized by an unusually high proportion of type 2 myocardial infarction (35% of post-TAVR ACS) and a very low proportion of ST-segment elevation myocardial infarction (STEMI) (less than 10% of post-TAVR ACS cases) [26]. These features are likely related to the advanced age and high comorbidity burden of TAVR recipients in the last 10 years. Second, although relatively rare, STEMI post-TAVR is associated with a particularly poor prognosis [26]. Finally, up to 65% of patients presenting with ACS post-TAVR do not receive coronary revascularization, and the lack of revascularization is obviously associated with an increased risk of MACCE. The low rate of coronary revascularization may be explained by the advanced age, the comorbidity burden, the high proportion of type 2 ACS, but also by potential coronary cannulation issues related to interactions between the transcatheter valve stent and coronary ostia [26].

Thus, one study focused on post-TAVR STEMI and compared the characteristics to those of all-comers STEMI (without TAVR history) [27]. This study led to the following findings:

- All STEMI management performance indicators were significantly worse in the population with a history of TAVR, with a 33% longer door-to-balloon time, but also a significantly longer procedure time, fluoroscopy time, and higher contrast volume and dosimetry level, reflecting increased procedural complexity.
- The PCI failure rate was four times higher in the population of patients with a history of TAVR (16% versus 4%), with particularly high rates of coronary cannulation failure (6%) and lesion crossing failure (5%).
- Revascularization failure was an independent predictor of poor outcomes.
- In addition to the classical atherothrombotic mechanism, other alternative mechanisms were involved such as coronary embolism (complicating the TAVR procedure itself, or related to TAVR valve thrombosis), late migration of the TAVR valve resulting in delayed coronary occlusion (particularly with self-expanding valves), or restenosis/thrombosis of stents implanted in the work-up pre-TAVR.

All these characteristics make post-TAVR ACS a unique population, complex to manage and with poor outcomes. Coronary re-access is a central issue, whose resolution will contribute to the final expansion of TAVR indications.

## 8. Coronary Access after TAVR

**Figure 4:**
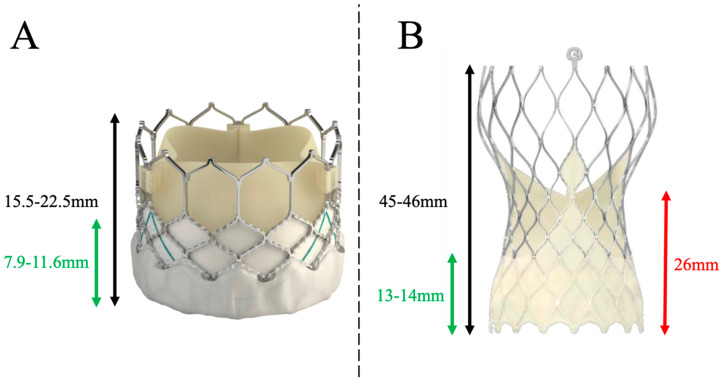
Shape and size of SAPIEN 3 (**A**) and Evolut R/PRO (**B**) transcatheter heart valves. Expanded height (black), commissural height (red), and skirt height (green).

Preliminary data on coronary canulation post-TAVR providing from case reports and small series have highlighted the highly variable success rate of selective coronary injection, ranging from 50% to 100% [2]. In 2018, Yudi et al. [28] proposed an algorithm to successfully perform post-TAVR coronary cannulation, depending not only on the implanted transcatheter heart valve but also on the position of the commissures regarding coronary ostia. The authors recommended the systematic use of 6-Fr catheters and a left radial or femoral approach for self-expanding supra-annular EVOLUT valves. Indeed, the design of the TAVR prosthesis (Figure 4) and its implantation technique (implantation depth, commissural alignment) will critically determine the feasibility of coronary artery cannulation after TAVR.

**Figure 5:**
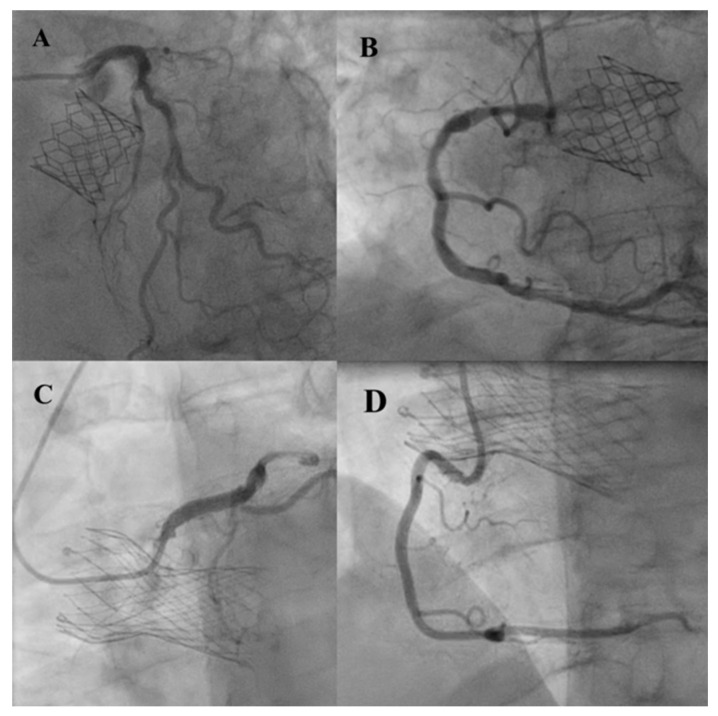
Coronary cannulation through Sapien 3 (**A**,**B**) and Evolut (**C**,**D**) transcatheter heart valves.

When a SAPIEN 3 valve is implanted, the stent of the prosthesis covers the coronary ostia in 40% to 50% of cases [29,30], compared with 100% of cases with the EVOLUT valve [31] (Figure 5). A study based on systematic post-TAVR CCTA showed that coronary ostia were in an unfavorable position (coronary ostium below the upper part of the skirt or in front of the commissures of the TAVR valve) in 8% (right coronary) to 15% (left coronary) of cases with SAPIEN 3 valves, compared with 25% (right coronary) to 35% (left coronary) of cases with EVOLUT valves [32]. Finally, the authors of the RE-ACCESS study performed systematic coronary angiography post-TAVI in a population comprising one-third EVOLUT valve, one-third SAPIEN 3 valve and one-third ACURATE NEO valve [33]. They observed a cannulation failure rate of 7.7% (23 patients in total, EVOLUT valve for 22 patients). Independent factors predicting coronary cannulation failure were EVOLUT valve, implantation depth, and the ratio of the TAVI implantation width to sinus of Valsalva width.

**Figure 6:**
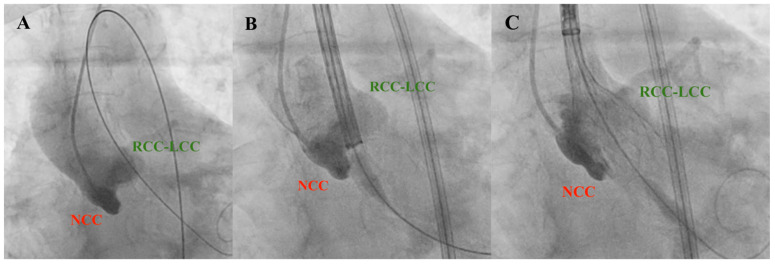
Valve deployment using the cusp-overlap view technique. NCC, non-coronary cusp; RCC, right coronary cusp; LCC, left coronary cusp. (**A**) Initial aortography; (**B**) Transcatheter valve approach; (**C**) Transcatheter valve deployment.

In order to facilitate coronary ostia catheterization after EVOLUT valve implantation, which will always have to be performed through the stent struts of the prosthesis, a new implantation technique based on commissural alignment enabled by a cusp-overlap view implantation has been developed and rapidly popularized [34]. This implantation technique reduces the rate of post-TAVI pacemaker implantation by minimizing implantation depth and is thought to facilitate coronary cannulation by aligning the commissures of the native valve with those of the TAVI valve (Figure 6). However, asymmetry of the aortic cusps is not uncommon (particularly in bicuspid valves), and an eccentric position of the coronary ostium is reported in 28% of right coronaries and 6% of left coronaries [35]. Consequently, the commissural alignment technique may not be sufficient in some cases, and it has been proposed to perform an alignment based on a “coronary artery overlap view” rather than a “cusp overlap view”, which enables one of the TAVR valve commissures to be positioned equidistant from the two coronary ostia, guaranteeing the best possible access to both coronaries.

## 9. Limitations

Most of published studies are observational by design, and randomized trials focusing on coronary revascularization pre-TAVR are expected. Data regarding acute coronary syndrome and coronary re-access after TAVR are currently limited to case reports and series of cases. TAVR recipients are now young patients with low surgical risk and long-life expectancy. The issue of coronary access post-TAVR will therefore become increasingly important in the coming years.

## 10. Conclusions and Future Directions

CAD is one of the most common comorbidities among TAVR patients. Coronary revascularization of thigh coronary stenosis located on proximal coronary segments is currently recommended during the work-up pre-TAVR. However, uncertainties remain regarding the clinical benefit of pre-TAVR coronary revascularization, and further studies will help to better identify patients and coronary lesions that should be revascularized prior to TAVR. Finally, the management of post-TAVR coronary events is an emerging issue that will become increasingly important. The fact that a significant number of post-TAVR ACS patients will be managed in non-TAVR centers underlines the critical importance of training all interventional cardiologists in techniques that can facilitate coronary artery cannulation after TAVR.

## Figures and Tables

**Table 1:** Complex coronary artery disease excluded from randomized trial.

Evolut Low Risk	Unprotected left main coronary artery, or
	Multivessel coronary artery disease with a SYNTAX score >22
PARTNER 3	Unprotected left main coronary artery, or
	SYNTAX score >32 (in the absence of prior revascularization), or
	Heart Team assessment that optimal revascularization cannot be performed

## Data Availability

Not applicable.

## Abbreviations

ACS	acute coronary syndrome
CAD	coronary artery disease
CCTA	cardiac computed tomography angiography
FFR	fractional flow reserve
iFR	instantaneous wave-free ration
MACCE	major adverse cardiovascular and cerebrovascular event
PCI	percutaneous coronary intervention
SAVR	surgical aortic valve replacement
TAVR	transcatheter aortic valve replacement
